# Software technical debt prediction based on complex software networks

**DOI:** 10.1371/journal.pone.0323672

**Published:** 2025-06-09

**Authors:** Bo Jiang, Jiaye Cen, Erluan Zhu, Jiale Wang

**Affiliations:** School of Computer Science and Technology, Zhejiang Gongshang University, Hangzhou, Zhejiang, China; National Institute of Technology, India (Institute of National Importance), INDIA

## Abstract

Technical debt prediction (TDP) is crucial for the long-term maintainability of software. In the literature, many machine-learning based TDP models have been proposed; they used TD-related metrics as input features for machine-learning classifiers to build TDP models. However, their performance is unsatisfactory. Developing and utilizing more effective metrics to build TDP models is considered as a promising approach to enhance the performance of TDP models. Social Network Analysis (SNA) uses a set of metrics (i.e., SNA metrics) to characterize software elements (classes, binaries, etc.) in software from the perspective of software as a whole. SNA metrics are regarded as a compensation of TD-related metrics used in the existing TDP work, and thus are expected to improve the performance of existing TDP models. However, the effectiveness of SNA metrics in the field of TDP has never been explored so far. To fill this gap, in this paper, we propose an improved software technical debt prediction approach. First, we represent software as a Class Dependency Network, based on which we compute the value of a set of SNA metrics. Second, we combine SNA metrics with the TD-related metrics to create a combined metric suite (CMS). Third, we employ CMS as the input features and utilize seven commonly used machine learning classifiers to build TDP models. Empirical results on a publicly available data set show that (i) the combined metric suite (i.e., CMS) can indeed improve the performance of existing TDP models; (ii) XGBoost performs best among the seven classifiers, with an ${\text{F}}_{2}$ value of 0.77, an *MI* ratio of approximately 0.10, and a *recall* close to 0.87. Furthermore, we also reveal the relative effectiveness of different metric combinations.

## 1 Introduction

Technical Debt (TD) is used to describe the quality compromises that can generate short-term benefits but have negative impacts on software evolution [1, 2]; it reflects the additional cost required for software maintenance. If TD is left unchecked, then it may lead to software that is difficult to maintain, or even *technical bankruptcy* [3]. Thus, there is an urgent need for effective methods to predict the potential TD in software, as TD prediction (TDP) is crucial for the long-term maintainability of software. Specifically, TDP can help project managers identify potential TD problems in software early on and refactor them before they accumulate and develop, thereby avoiding excessive debt accumulation and reducing maintenance costs.

In the literature, many machine-learning based TDP models have been proposed. They used software metrics that are related to TD (e.g., object-oriented (OO) metrics [4], code smells related metrics [5], software change related metrics [6], and software refactoring related metrics [7]) as input *features* and employed machine learning techniques (e.g., *random forests*, *linear regression*, and *decision trees*) to build models for TDP. Thus, developing and utilizing more effective metrics to build TDP models is considered a promising approach to enhance the performance of TDP models.

Social network analysis (SNA) [8] is a research methodology that explores social structures by analyzing the interactions between individuals, organizations, and groups. It aims to understand the structure, evolution, and impact of social networks. When performing the SNA, they should first represent the subject to be analyzed as a dependency network, where individuals, organizations, and groups are nodes, and their interactions are edges. Then, they will employ a set of metrics (called SNA metrics) to characterize the nodes in the dependency network.

SNA metrics have been introduced into the field of software defect prediction (SDP), where researchers represented software as dependency networks (e.g., class dependency networks), where classes/binaries are nodes, relationships between classes (e.g., *method class*, *inheritance*, and *implements*) are edges, and employed SNA metrics to characterize the class complexity. These SNA metrics of classes are used as *features* to build SDP models. Empirical results show that SNA metrics are effective metrics to improve the performance of SDP models. For example, Zimmermann and Nagappan [9] found that SNA metrics can significantly improve the performance of SDP models. Nguyen *et al*. [10] replicated the work of Zimmermann and Nagappan [9], and confirmed that the combined metric set of SNA metrics and code metrics does help improve the performance of within-project SDP models. SNA metrics are computed from the dependency networks as a whole, and thus they are greatly different from the metrics used in the existing TDP work (we refer to them as *TD-related metrics*), such as object-oriented (OO) metrics [4], code smells related metrics [5], software change related metrics [6], and software refactoring related metrics [7], which are mainly computed from the local structure of software. In this sense, SNA metrics can be used to enrich the TD-related metrics used in the existing TDP work, and help to more comprehensively describe the code characteristics of software; we can expect that combining SNA metrics with TD-related metrics can improve the performance of existing TDP work. However, the effectiveness of SNA metrics in the field of TDP has never been explored so far.

To fill this gap, in this paper, we propose an improved software technical debt prediction approach – TDPSN (software technical debt prediction based on complex software networks). First, TDPSN represents software as a Class Dependency Network (CDN) [11–15], based on which TDPSN computed the value of a set of SNA metrics. Second, TDPSN combines SNA metrics with the TD-related metrics to create a combined metric suite (CMS), which is composed of eight categories of metrics. Third, TDPSN employs CMS as the input features and utilizes seven commonly used machine learning classifiers (i.e., Logistic Regression, Naive Bayes, Decision Tree, K-Nearest Neighbors, Support Vector Machine, Random Forest, and XGBoost) to build TDP models. Empirical results on a publicly available data set show that i) the combined metric suite (i.e., CMS) can indeed improve the performance of existing TDP models, with five out of the seven classifiers showing improvement in ${\text{F}}_{2}$-measure and Module Inspection (*MI*) ratio; ii) XGBoost (XGB) emerges as the best classifier among the seven classifiers, with an ${\text{F}}_{2}$ value of 0.77, an *MI* ratio of approximately 0.10, and a *recall* close to 0.87, which means that by detecting 10% of potential high-TD modules, we can identify approximately 87% of all true high-TD modules. Furthermore, we also reveal the relative effectiveness of different metric combinations, such as *size* vs. *evolution*, *complexity* vs. *evolution*, and *coupling* vs. *cohesion*.

Our main contributions include:

- We are the first to introduce SNA metrics into the TDP field, revealing that the combined metric suite of SNA metrics and TD-related metrics can significantly improve the performance of TDP models.
- We find that among the seven commonly used machine-learning classifiers, XGBoost performs best for TDP if we use CMS as the input features.
- SNA metrics are composed of two categories – GN metrics and EN metrics; TD-related metrics are composed of six categories – size-related, evolution-related, duplication-related, complexity-related, coupling-related, and cohesion-related metrics. We reveal that i) “TD-related metrics + GN metrics" outperforms “TD-related metrics + EN metrics", and ii) “size-related metrics + SNA metrics" outperforms other metric combinations.

The rest of this paper is organized as follows: Section 3 describes our research questions, as well as the projects, metrics, models, experimental settings, and evaluation measures used in the study. Section 4 presents the experimental results corresponding to the research questions. Section 5 discusses the implications inferred from our results and potential threats to the validity of our findings. Finally, Section 6 concludes this paper.

## 2 Related work

The existing research on TDP can be mainly divided into two categories: one is based on time series models (ARIMI), however, ARIMI has deficiencies in long-term TDP performance. Therefore, another category of research based on machine learning techniques has emerged. This type of research mainly constructs TDP models by combining various software metrics related to TD.

**Time series techniques for technical debt forecasting:** Time series (ARIMA) models were initially utilized in TDP. However, while ARIMA models demonstrate strong performance in predicting short-term TD evolution, they do not yield optimal results for long-term prediction (greater than 8 weeks) [16]. Therefore, machine learning techniques have begun to be widely applied in TDP, as machine learning algorithms possess the capability to handle irrelevant features and support complex relationships between variables as well as tolerate noise.

**Machine learning techniques for technical debt forecasting:** The study conducted by Chug and Malhotra [17] utilized object-oriented (OO) metrics as predictors to forecast the future maintainability of software. The study concluded that the genetic adaptive learning model outperformed other models, based on a comparison of various common machine learning techniques. Code smells are considered an important metric in the field of TDP. According to Ref. [18], research has been summarized on the use of machine learning algorithms to predict code smells. They assert that ML algorithms deliver strong performance, with random forest being identified as one of the best algorithms. Furthermore, numerous other software quality metrics such as software change proneness and software refactoring have been applied to TDP. Tsoukalas *et al*. [19] argued that while machine learning (ML) technology has been widely applied to build Technical Debt Prediction (TDP) models, its ability to identify and predict High-TD modules is still subject to further investigation. They constructed a TDP model by extracting 18 software quality metrics related to TD from 25 open-source Java projects, and the experimental results ultimately demonstrated that superior classifiers can effectively predict High-TD modules.

**Network metrics:** However, the existing research on TDP has not achieved the desired results, especially in the development and utilization of metrics. Currently, the TD-related metrics shown in Table 1 are basically obtained through static source code analysis. Moreover, the dependency relationships between software modules are a rich source of information. Specifically, data dependencies, which define how data is passed between different parts of the code, and call dependencies, which show which functions or methods call others, can be directly extracted from the source code. Additionally, in the context of analyzing these dependencies through Social Network Analysis (SNA), various types of network metrics play crucial roles. Centrality Metrics, such as degree centrality which measures the number of direct connections a module has in the dependency network, and betweenness centrality that identifies components that lie on the shortest paths between other components, help in pinpointing components with disproportionate influence over dependency flows. Path Analysis, like calculating the average shortest path in the dependency network, can reveal latent debt propagation channels, showing how issues in one module might spread through the system. Modularity Metrics are used to quantify the architectural erosion caused by dependency sprawl, indicating how well - defined and separated different parts of the software architecture are despite the existing dependencies [20].

**Table 1:** Selected projects.

Metric type	Metric	Description
Reliability Metrics	bugs [23, 24]	Total number of bug issues of a project
Security Metrics	vulnerabilities [23, 24]	Total number of vulnerability issues of a project
Maintainability Metrics	code_smells [23, 25]	Total number of code smell issues of a project
Size metrics	comment_lines [24]	Number of lines
Size metrics	ncloc [23]	Number of physical lines of a project
Coverage Metrics	uncovered_lines [24]	Number of lines of code of a project
Duplication Metrics	duplicated_blocks [24, 26]	Number of duplicated blocks of lines of a project
Complexity Metrics	complexity [27]	The Cyclomatic Complexity of a project
Complexity Metrics	AMC [28]	Average Method Complexity
Complexity Metrics	WMC [27]	Weighted Methods per Class
Complexity Metrics	DIT [27]	Depth of Inheritance Tree
Complexity Metrics	NOC [27]	Number of Children
Complexity Metrics	RFC [27]	Response for a Class
Cohesion Metrics	LCOM [27, 29]	Lack of Cohesion in Methods
Cohesion Metrics	LCOM3 [30]	Lack of Cohesion in Methods
Cohesion Metrics	CAM [29]	Cohesion Among Methods
Coupling Metrics	CBO [27, 29]	Coupling Between Objects
Coupling Metrics	Ca [31]	Afferent Coupling
Coupling Metrics	Ce [31]	Efferent Coupling
Coupling Metrics	CBM [27]	Coupling Between Methods
Coupling Metrics	IC [27]	Inheritance Coupling
Other Metrics	NPM [30]	Number of Public Methods
Other Metrics	DAM [32, 33]	Data Access Metric
Other Metrics	MOA [32, 33]	Measure of Aggregation

Social Network Analysis (SNA) metrics have been extensively utilized in the field of software engineering, particularly in software defect prediction (SDP). Zimmermann and Nagappan [21] extracted a set of dependency network metrics, namely Social Network Analysis (SNA) metrics, from the dependency network of software modules through Social Network Analysis. They believed that SNA metrics could better locate defects in software by capturing the dependency relationships within the software system. And through systematic experiments, they proved that SNA metrics could effectively improve the prediction performance of the defect prediction model (by 10%). This conclusion was supported by the repetitive experiments conducted by the Nguyen team [10]. They found that the combined use of SNA metrics and code metric metrics could effectively enhance the accuracy of defect prediction within projects. Gong *et al*. [22] through a case study involving 9 open-source software across 30 versions, examined the performance of SNA metrics in cross-project SDP and confirmed their effectiveness. Furthermore, they observed varying impacts on model performance from ego network (EN) and global network (GN) metrics within SNA metrics.

## 3 Experiment setup

### 3.1 Research question

In this work, we will investigate three research questions (i.e., RQ1 to RQ3). These research questions aim to investigate the impact of SNA metrics on TDP models. In RQ1, we will examine whether the combined metric suit (CMS) can enhance the performance of the existing TDP model. Following an assessment of the effectiveness of SNA metrics in improving the TDP model, we will re-evaluate the performance of each classifier in the TDPSN models in RQ2 to identify the most suitable classifier for the TDPSN model. In the final stage of RQ3 and RQ4, we will utilize the best classifier obtained from RQ2 to investigate the impact of different combinations of metrics (TD-related and SNA-related metrics) on the performance of the TDPSN models.

#### 3.1.1 RQ1: Can SNA metrics improve the performance of TDP models?

As mentioned in Section 1, SNA metrics are built from software as a whole and thus can provide additional information of the software that TD-related metrics cannot capture. However, to the best of our knowledge, there has been no research work on the application of SNA metrics to build TDP models so far. Thus, in RQ1, we will investigate whether the introduction of SNA metrics can enhance the performance of TDP models.

#### 3.1.2 RQ2: Which classifier performs best on TDPSN models?

Tsoukalas *et al*. [19] found that Random Forest is the best classifier on TDP when using TD-related metrics as input features; but XGBoost and SVM also have competitive performance. In this paper, a combined metric suite, CMS, is introduced. Thus, in RQ2, we will reevaluate the performance of the three classifiers when using CMS as input features and find the best classifier for TDP.

#### 3.1.3 RQ3: Are SNA metrics more effective than TD metrics?

In RQ1, this paper verified that the combination of SNA metrics and TD-related metrics can improve the performance of the TDP model to a certain extent. However, the predictive ability of SNA metrics themselves for TD and the contributions of different categories of metrics to the TD identification ability still lack research. To address the above issues, this paper classifies different SNA metrics and TD - related metrics, and adopts certain combination and feature selection strategies to examine the impacts of different categories of metrics on the performance of the TD identification model respectively.

#### 3.1.4 RQ4: Can diverse metric combinations influence the performance of TDPSN models?

Gong *et al*. [22] discovered that Ego network (EN) and Global network (GN) metrics in SNA metrics have varying impacts on the performance of SDP models. In some cases, models trained with GN metrics exhibited superior performance. Furthermore, we categorized TD-related metrics into six categories in our study. All TD-related metrics and SNA metrics used in our research are reported in Section 3.3. Therefore, in RQ3, we will explore the impact of different metrics combinations on the performance of TDP models.

### 3.2 Subject projects

We conduct our experiments on the 25 same Java projects as Tsoukalas *et al*. [19]. These Java projects cover a wide range of diverse fields and are representative. For example, Arduino represents the Internet of Things and embedded systems, while we have chosen Pdfbox and Libgdx for the PDF and game development fields respectively. The diversity of the projects can enhance the stability and generalizability of our research results. Table 2 details the 25 projects, including their names, descriptions, version information, and code line counts (Loc). The code line counts range from 7K to 482K, reflecting the varied project and code sizes we examined.

**Table 2:** Selected projects.

Project	Description	Loc
arduino	Physical open-source electronics platform	27K
arthas	Java Diagnostic tool	28K
azkaban	Workflow manager	79K
cayenne	Powerful ORM framework for Java	348K
deltaspike	Collection of portable CDI extensions	146K
exoplayer	Open-source media player for Android	155K
fop	Print formatter driven by XSL formatting objects	292K
gson	Java object to relational mapping framework	25K
javac	Java compiler for translating source code to bytecode	23K
jclouds	Multi-cloud toolkit for cloud management	482K
Joda-time	Date and time library with a comprehensive API	86K
libgdx	Cross-platform game development framework	280K
maven	Build automation tool for managing Java projects	106K
mina	Network application framework for Java	35K
nacos	Service discovery and configuration management platform	60K
opennlp	Machine learning toolkit for natural language processing	93K
openrefine	Tool for cleaning and analyzing messy data	69K
pdfbox	Java library for working with PDF documents	213K
redission	Java client for Redis with distributed objects	133K
RxJava	Library for composing asynchronous programs	310K
testng	Testing framework	85K
vassonic	Lightweight WebView solution for Android	7K
wss4j	Library for implementing web services security standards	136K
xxl-job	Distributed task scheduling framework for Java	9K
zaproxy	Security tool	187K

### 3.3 Metric suites

In this section, we briefly outlined the TD metrics and SNA metrics used in our study, as well as several combined metrics derived from them.

#### 3.3.1 TD metrics.

We use the TD (TD-related) metrics by Tsoukalas *et al*. [19]. We refer to their classification of the TD index in subsequent work [34] and categorize the 18 TD metrics used in our study into 6 categories, as shown in Table 3. Different metric categories represent different information about software projects. Size metrics measure the size and complexity of a project, for example, Total Methods and Total Variables can represent the number of methods and variables in a codebase. Evolution metrics can provide information about the evolution of a codebase by analyzing its commit history and development activities. Duplication metrics assess the reusability and maintainability of a codebase by measuring the proportion of duplicate code lines in it. Complexity metrics provide information about the complexity of a codebase by measuring its structural and design complexity. Coupling metrics assess the coupling degree of a codebase by analyzing the dependency relationships between modules in it. Cohesion metrics measure the cohesion of a codebase by assessing the functional relatedness and consistency of modules within it. The SM (Simple Combined) metrics in the Combined Metrics category are a set of metrics that were simply combined from the Duplication Metrics, Complexity Metrics, Coupling Metrics and Cohesion Metrics categories to meet the research requirements of RQ3 in Section 4.

**Table 3:** TD metrics used in our study.

Metric group	Metrics	Metrics	count
TD metrics	Size	Comment lines density, Ncloc,	5
		Max nested blocks, Total methods,	
		Total variables	
	Evolution	Commits count, Code churn avg,	7
		Contributors count, Contributors	
		experience, Hunks count, Total	
		refactorings, Issue tracker issues	
	Duplication	Duplicated lines density	1
	Complexity	DIT, WMC, RFC	3
	Coupling	CBO	1
	Cohesion	LCOM	6
Combined	SM	Duplication + Complexity + Coupling+ Cohesion	6


#### 3.3.2 SNA metrics.

Social network analysis (SNA) is a method used to investigate interpersonal connections and information dissemination. It has been widely applied in the field of software engineering as well. SNA simplifies the relationships between software modules into a network model, with nodes representing elements within the software and edges denoting their relationships, including data and call connections. By conducting social network analysis on this relationship network, various SNA metrics can be derived, which can be further categorized into EN (ego network) metrics and GN (Global network) metrics. The complete set of 64 SNA metrics along with their combined metric suit (CMS), including TD metrics, are presented in Table 4.

**Table 4:** SNA metrics used in our study.

1Metric group	Metrics	Metrics	count
SNA Metrics	EN	Size {in, out, un}, Ties {in, out, un},	40
		Pairs {in, out, un}, Density {in, out, un},	
		WeakComp {in, out, un}, nWeakComp {in,	
		out, un}, 2StepReach {in, out, un},	
		ReachEfficency {in, out, un}, Brokerage	
		{in, out, un}, nBrokerage {in, out, un} ,	
		EgoBetween {in, out, un}, nEgoBetween	
		{in, out, un}, EffSize(ego), Efficiency(ego),	
		Constraint(ego), Hierarchy(ego)	
	GN	Degree, Eigenvector, power, Closeness,	24
		Betweenness, Information, dwReach,	
		EffSize, Efficiency, Constraint, Hierarchy	
Combined Metrics	SM	Commits count, Code churn avg, Contributors count,	46
		Contributors experience, Hunks count, Issue tracker issues,	
		CBO, WMC, DIT, RFC, LCOM, Max nested blocks, Total methods, Total variables, Total variables, Total refactorings,	
		Ncloc, Hierarchy, EgoBetween(in), nWeakComp(out),	
		pWeakComp(out), nBroker(out), EgoBetween(out),	
		nEgoBetween(out), Density(un), nWeakComp(un),	
		Broker(un), nBroker(un), nEgoBetween(un), nOutdeg,	
		nIndeg,Eigenvec, nEigenvec, inCloseness,	
		outCloseness,Betweenness, nBetweenness,InfoCent,	
		nOutdwReach, Constraint(g), Hierarchy(g), ReachEffic(out), Density(out), Density(in), pWeakComp(in), ReachEffic(in)	
	SNA	EN metrics+GN metrics	64
	CMS	TD metrics+SNA metrics	82
	OM	Duplication + Complexity+ Coupling+ Cohesion	6


**Ego network (EN) metrics:** EN is a network that focuses on examining a specific node and its connections and relationships with other nodes [17]. Within the self-network, “in," “out," and “un" represent the different types and directions of connections between a node and its neighboring nodes. For instance, “in" reflects the benefits and influence of the node itself within the network, such as contributions or support from other nodes to the focal node. On the other hand, “out" represents the contribution or influence of the focal node to its neighbor nodes, indicating its support or influence on other nodes. The term “un" refers to the bidirectional connection between self-nodes and neighboring nodes, without a clear direction. This indicates that there is mutual interaction and relationship between self-nodes and neighboring nodes, and the flow of information and resources between them is bidirectional. In our study, we utilized all three types of self-networks.

**Global network (GN) metrics:** GN refers to the structure and characteristics of the entire network system. Global network metrics are used to describe and analyze the properties and characteristics of the entire network, such as overall scale, average path length, network density, and clustering coefficient. These metrics can help researchers understand the overall structure of the entire network, the importance of central nodes, the efficiency of information diffusion, and the structures that exist in the network.

Finally, we combined the 18 TD metrics with 64 SNA metrics to obtain a combined metric suite (CMS) containing 82 metrics.

#### 3.3.3 Combined metrics.

We explore two different methods of combining TD and SNA indicators to determine if their amalgamation outperforms individual metrics. The CMS indicator merges TD and SNA metrics without discarding any. In contrast, the OM indicator combines a select set of TD metrics (Duplication, Complexity, Coupling, and Cohesion) due to their limited numbers (ranging from 1 to 3). We opt for this grouping to prevent information scarcity from impacting our results. The SM indicator, derived through a simple filtering approach, aims to uncover stronger signals masked by other metrics in the amalgamated set. Drawing insights from prior studies [19, 22], we employ two distinct filtering techniques for feature selection:

**Univariate logistic regression analysis:** To ascertain the relationship between chosen metrics and predicting high-TD modules, we apply univariate logistic regression. This method, extensively used in software engineering research [21], focuses on establishing significant statistical links between individual metrics and high-TD classes. We set the significance threshold at a = 0.05. Metrics with p values below 0.05 are deemed statistically significant for high-TD classes, while those above 0.05 are pruned from further analysis due to their insignificance.

**VIF Variance Inflation Factor:** To refine feature selection and mitigate multicollinearity, we implement VIF collinearity checks. By regressing each predictor against others and calculating the coefficient of determination (${\text{R}}^{2}$), the VIF formula is applied. As per existing research [35], VIF values between 1-5 denote moderate correlation, while 5-10 suggest potential multicollinearity. Adhering to conventions from similar studies [22], we set the cut-off at 10, focusing solely on features with VIF values < 10.

Initially, univariate regression is conducted on the base feature set (CMS), leading to the elimination of 6 features with no significant impact on the target variable. Subsequently, VIF values are calculated for the remaining set to weed out highly collinear metrics. Following these filtering steps, the final SM feature set comprises 46 indicators, indicating the removal of 30 features. We present all 46 selected indicators from the refined SM feature set in Table 4.

### 3.4 Data pre-processing

We utilized the dataset employed by Tsoukalas *et al*. [19] in their study as the foundation for our research. This dataset consists of a table with 18,857 rows (representing software modules) and 19 columns. The first 18 columns each correspond to a software quality metric related to TD, as outlined in Table 3. At the end of the table are the Max-Ruler values (A definition by Amanatidis *et al*. [36], i.e., whether the module is a high-TD module). "1" and "0" are used to distinguish whether a module is a high-TD module, "1" represents a high-TD module, and "0" represents a no high-TD module. In subsequent research, we will refer to this dataset as the TD dataset.

In this work, the 40 EN metrics and 24 GN metrics for each module are extracted from the Class Dependency Network (CDN) [11–15] of 25 Java programs using the UCINET tool [20]. The specific metrics are presented in Table 4. CDN is a directed network (CDN = (*V*, *E*)), where each node ${\text{v}}_{c}\in V$ represents a class (or interface) *c* in the program, and the edge set E represents the class dependencies. Each edge $<{\text{v}}_{i}$, ${\text{v}}_{j}>$ (${\text{v}}_{i}$, ${\text{v}}_{j}\in V$) indicates the dependency relationship from class ${\text{v}}_{i}$ to class ${\text{v}}_{j}$.

There are a total of 9 types of dependencies between classes [11–15]:

- Local variable (LV): Class *i* contains a variable of class *j*.
- Global variable (GV): Class *i* contains a field of class *j*.
- Inheritance (INH): Class *i* inherits class *j*, adding new functionality through the inheritance relationship indicated by the ‘extends’ keyword.
- Interface implementation (II): Class *i* implements the functionality of interface *j*.
- Parameter type (PT): Class *i* contains at least one method with a parameter of class *j*.
- Return type (RT): Class *i* contains a method with a return type of class *j*.
- Instantiates (INS): Class *i* instantiates objects of class *j*.
- Access (AC): Class *i* has at least one method accessing fields of class *j*.
- Method call (MC): Class *i* has at least one method calling a method on an object of class *j*.

**Fig 1:**
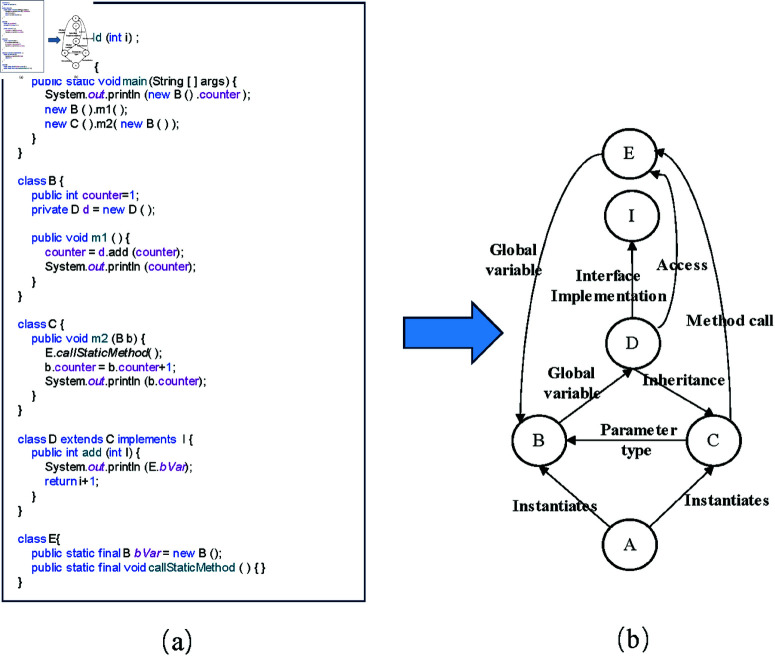
An illustrative Java code snippet and its Class Dependency Network (CDN). ( **a**) A code snippet to illustrate the CDN more clearly. ( **b**) The CDN based on the modified code snippet.

Fig 1a presents a code snippet to illustrate the CDN more clearly, and Fig 1b depicts the CDN based on the modified code snippet. Similar to previous studies, the nodes in CDN represent classes, and the edges represent dependencies between classes. However, in our case, we further consider three additional types of dependencies: “instantiates,” “access” and “method call.” We also differentiate the “aggregation” relationship into “local variable” and “global variable.” As shown in Fig 1b, the code snippet in Fig 1a demonstrates various dependencies between classes: global variables (B→D, E→B), inheritance (D→C), interface implementation (D→I), parameter type (C→B), instantiation (A→B, A→C, D→C), and access (D→E).

Then, we combined the SNA dataset with the TD dataset to obtain a final dataset containing 18,857 rows and 83 columns. Following the suggestion of Tsoukalas *et al*. [19], we conducted missing value treatment on the dataset and removed modules containing missing values. This operation resulted in a new dataset consisting of 17,646 modules. As the independent variables in the dataset exhibited non-normal distribution and extreme values, we utilized *Local Outlier Factor* (LOF) [37] to enhance model performance. Post outlier removal, the dataset contained 13,515 modules, with 4,131 modules identified as outliers and subsequently removed. However, experimental findings indicated that this process did not enhance model performance; instead, it resulted in a decrement. This outcome may be attributed to the excessive removal of modules (23% of the total) compared to the approach by Tsoukalas *et al*. [19], leading to reduced data dimensionality and information loss. Given the inherent disparities between the datasets and metrics, we opted to solely eliminate modules with outliers in the TD dataset. This procedure led to the removal of 739 modules, resulting in a final dataset of 16,907 rows and 83 columns. For future reference, we denote this dataset as the CMS (combined metric suit) dataset.

### 3.5 Model building

#### 3.5.1 Model selection.

We employed seven commonly used machine learning classifiers in the field of TDP: Logistic Regression (LR), Naive Bayes (NB), Decision Tree (DT), K-Nearest Neighbors (KNN), Support Vector Machine (SVM), Random Forest (RF), and XGBoost (XGB). These classifiers have been widely used in other similar studies, and among them, the RF classifier is generally considered to be an excellent model for TDP [38].

- RF: An ensemble learning framework based on Bootstrap aggregation. It constructs multiple diverse decision trees through feature subsampling and integrates the prediction results using a voting mechanism. By reducing the correlation of a single tree, it improves the generalization performance, but it has relatively low computational efficiency for high-dimensional sparse data.
- LR: A generalized linear classification model that maps a linear combination to a probability output through the Sigmoid function, and it is suitable for binary classification tasks. It can be extended to multi-classification scenarios through the One-vs-Rest strategy. Its decision boundary has a linear characteristic, and it is sensitive to the multicollinearity among features.
- KNN: A lazy learning algorithm based on spatial similarity. In the training stage, it only stores the entire sample set. During inference, it retrieves the K nearest neighbor samples through a distance metric (such as Manhattan distance) and determines the class membership using a majority voting mechanism. The performance of the model is significantly affected by the data distribution density and the curse of dimensionality.
- NB: A probabilistic model based on Bayes’ theorem and the assumption of feature conditional independence. It calculates the posterior classification probability through prior probability and likelihood estimation. Although it performs robustly in text classification, there will be probability estimation bias in scenarios where the feature correlation is strong.
- SVM: A margin classifier based on structural risk minimization. It maps the low-dimensional inseparable data to a high-dimensional feature space through the kernel trick and searches for the maximum margin hyperplane to achieve classification. It is sensitive to outliers, and the selection of the kernel function directly affects the model complexity.
- XGBoost: An optimized ensemble algorithm based on gradient boosting decision trees. It accelerates the convergence of the loss function through a second-order Taylor expansion and introduces a regularization term to control the model complexity. It supports parallel computing and missing value handling and shows significant advantages in various prediction tasks.
- DT: A white-box model that adopts a tree structure. It recursively selects the optimal feature to divide nodes through information gain or Gini coefficient until the purity threshold is reached. It is prone to overfitting and often requires pruning optimization, but it has the natural ability of feature selection and the advantage of visual interpretation.

#### 3.5.2 Model configuration.

To assess classifier performance, we implemented a stratified training-validation-test pipeline while preserving the original high-TD:not-high-TD class ratio (1:15). The dataset underwent an 80%:20% split, with the larger portion used for model development (including 3×10 stratified cross-validation) and the smaller held out for final evaluation on unseen data. Stratification ensured class distribution consistency across partitions, critical for realistic TDP scenario modeling.

Starting with the validation phase, 3×10 repeated stratified cross-validation strategy was adopted. Each 10-fold split maintained class ratios, with SMOTE [39] applied only to training folds during each iteration to address class imbalance while keeping test folds. This process generated 30 model iterations (3 repetitions × 10 folds) to mitigate sampling bias, with performance metrics averaged across runs. MinMax scaling normalized features (0–1 range) per fold to prevent variance dominance, applied only to training folds during each iteration to avoid data leakage. Grid search optimized hyperparameters using ${\text{F}}_{2}$-measure as the objective (detailed in 3.6), with tuning embedded within the cross-validation loop to ensure generalization.

After validation, fine-tuned classifiers were retrained on the full 80% training-validation set (with SMOTE and MinMax scaling) and evaluated on the held-out 20% test set—never exposed during development. This mimicked real-world deployment where models predict high-TD modules in new systems. Unlike the averaged cross-validation metrics, test phase results represent single-pass evaluations on the complete test set.For our experiments, we used the Python language and more specifically the scikitlearn ML library.

Hyperparameter tuning: To ensure that the model could fit the dataset, we used the ${\text{F}}_{2}$ metric as the objective function of the estimator and used the *Grid-Search* [40] method to find the optimal parameters for the classifier. To avoid overfitting, we used 3 × 10 stratified cross-validation for parameter selection.The hyperparameter adjustment spaces for each classifier are as follows:

- For Logistic Regression (LR), four types of regularization (l1, l2, elasticnet, and none) were tested to explore the balance between sparse constraints and the model’s generalization ability. For Linear Discriminant Analysis (LDA), the adaptability of three solvers, namely Singular Value Decomposition (svd), Least Squares (lsqr), and Eigenvalue Decomposition (eigen), was compared in terms of covariance estimation methods.
- The tuning of Decision Trees (DT) focused on the splitting criteria (“gini,” “entropy”) and tree depth limits (None, 2, 5, 10 levels). The aim was to control the model complexity while retaining the discriminative power of key features.
- The K-Nearest Neighbors (KNN) algorithm explored the trade - off between the sensitivity to local patterns and noise resistance by traversing the number of neighbors (from 1 to 10).
- The parameter space of Support Vector Machines (SVM) covered two core kernel functions, the linear kernel and the Radial Basis Function (RBF) kernel (“linear,” “rbf”). The candidate values of the regularization coefficient C were set as an exponential sequence (from 0.01 to 1000) to evaluate the modeling effects of soft-margin and hard-margin strategies on non-linear boundaries.
- Random Forest (RF) was optimized from three dimensions: the ensemble size (from 5 to 1000 trees), the maximum depth of a single tree (None, 2, 5, 10 levels), and the splitting criteria (“gini,” “entropy”). The goal was to enhance the diversity of base learners and suppress the risk of overfitting.
- For XGBoost, the focus was on adjusting the number of boosting trees (from 5 to 1000). The optimal number of iterations was dynamically determined through an early - stopping mechanism.

### 3.6 Performance evaluation metrics

To evaluate the performance of TDP models, we used four different performance metrics: precision, recall, ${\text{F}}_{2}$-measure, and module detection (*MI*) ratio. In addition to precision and recall, which are common performance metrics in the prediction field, ${\text{F}}_{2}$-measure and *MI* ratio are considered the two most important performance metrics for evaluating TD prediction models in other similar studies (e.g., Ref. [19]).

${\text{F}}_{2}$-measure: In practical TD prediction, accurate identification of minority classes (high-TD modules) is paramount, as missing any high-TD module may lead to substantial economic losses and systemic risks [19]. According to the recommendations of Tsoukalas *et al*. [19], the selection of models prioritized high-recall performance while maintaining reasonable precision. Traditional evaluation metrics often fail to balance this requirement effectively, whereas the F measure addresses this limitation by harmonizing precision and recall. By setting the relative importance coefficient β=2, more emphasis was placed on recall to ensure that the model detects all high-TD modules with maximum precision. For example, misclassifying low-TD modules as high-TD (false positives/ ${\text{FP}}_{S}$) primarily causes resource waste (e.g., unnecessary refactoring) without direct system failure consequences. In contrast, misclassifying high-TD modules as low-TD (false negatives/ ${\text{FN}}_{S}$) leads teams to overlook critical risks, potentially triggering exponential growth in maintenance costs. This demonstrates the asymmetric risk between ${\text{FN}}_{S}$ and ${\text{FP}}_{S}$ [19]. To evaluate the prediction capacity of minority classifiers, the ${\text{F}}_{2}$ measure was adopted as the primary performance metric, defined as:

$$precision=\frac{TP}{TP+FP}$$ (1)

$$Recall=\frac{TP}{TP+FN}$$ (2)

$${\text{F}}_{2}-measure=(1+\beta^{2})*\frac{Precision*Recall}{(\beta^{2}*Precision)+Recall}$$ (3)

Module inspection (*MI*) ratio: The *MI* ratio is defined as the ratio of modules predicted to be high TD to the total number of modules [41]. It refers to the percentage of modules that developers must check to find the number of high TD modules that the model can correctly identify, which is the recall efficiency. For example, for a model with a recall rate of 80% and an *MI* ratio of 10%, it means that we need to manually check 10% of the total number of modules (in other words, only check the modules that the model predicts to be high TD) to find 80% of the true high TD modules (TP). Such a model is more cost-effective than randomly checking modules. It is defined as follows:

MI=(TP+FP)/(TP+TN+FP+FN) (4)

Therefore, the combination of ${\text{F}}_{2}$-measure and *MI* ratio paints a perfect picture for TD prediction. They cover the accuracy and practicality of the model, which means that the model can efficiently and accurately detect all high-TD modules to the greatest extent possible.‘

To evaluate the enhancement effect of Social Network Analysis (SNA) metrics on technical debt prediction, we apply the Wilcoxon signed-rank test [42]. We independently analyze 30 paired observations derived from 3×10-fold cross-validation. Moreover, we correct the obtained p-values from the Wilcoxon-signed rank test with Bonferroni correction [43] to control for false positives. We do so to statistically quantify the number of datasets on which models trained on other metric families outperform models trained on TD metrics with a statistical significance (p-values < 0.05).

## 4 Results and analysis

In this section, the research results related to the research questions will be presented and discussed. For all RQ in Section 3.1, the baseline method in Section 3.5 is followed.

### 4.1 Can SNA metrics improve the performance of TDP models?

Table 5 presents a collection of performance metrics for the classifiers under evaluation, which underwent validation and testing using the training-validation-testing approach outlined in Section 3.5. Specifically, the “validation" column displays the outcomes achieved by each classifier during repeated hierarchical cross-validation (the validation stage). These results reflect the overall metrics, averaged over 3 repetitions of performance indicators with k = 10, and include corresponding standard deviation values. In contrast, the “test” column illustrates the performance metrics achieved by each evaluated classifier on a separate set (i.e., data that was not utilized during training/validation and represents 20% of the total data). For model performance evaluation, our main focus is on the ${\text{F}}_{2}$-measure and the *MI* ratio mentioned in Section 3.6.

**Table 5:** Evaluation results for all classifiers.

		$F_{2}$		Precision		Recall		MI	
	Model	Validation	Test	Validation	Test	Validation	Test	Validation	Test
TD	RF	**0.756 (0.030)**	0.766	0.573 (0.026)	0.553	**0.823 (0.043)**	**0.847**	0.087 (0.006)	0.092
	XGB	0.710 (0.042)	0.725	0.668 (0.033)	0.675	0.723 (0.053)	0.739	0.065 (0.006)	0.066
	LR	0.761 (0.023)	0.762	0.454 (0.026)	**0.553**	**0.917 (0.031)**	**0.926**	0.122 (0.008)	0.124
	SVM	0.750 (0.023)	0.760	0.433 (0.021)	**0.553**	**0.918 (0.037)**	**0.936**	0.128 (0.008)	0.130
	KNN	**0.728 (0.025)**	0.730	**0.447 (0.024)**	**0.443**	**0.866 (0.036)**	**0.872**	0.117 (0.008)	0.118
	NB	**0.670 (0.032)**	0.698	**0.424 (0.024)**	**0.401**	**0.785 (0.043)**	**0.857**	0.111 (0.007)	0.128
	DT	**0.651 (0.551)**	0.657	**0.511 (0.039)**	0.528	**0.700 (0.063)**	0.700	0.082 (0.007)	**0.080**
CMS	RF	0.749 (0.032)	**0.768**	**0.584 (0.030)**	**0.596**	0.807 (0.040)	0.828	**0.083 (0.006)**	**0.083**
	XGB	**0.740 (0.032)**	**0.749**	**0.698 (0.038)**	**0.713**	**0.752 (0.040)**	**0.759**	**0.065 (0.005)**	**0.064**
	LR	**0.767 (0.027)**	**0.767**	**0.482 (0.032)**	0.475	0.901 (0.031)	0.906	**0.113 (0.008)**	**0.114**
	SVM	**0.757 (0.024)**	**0.764**	**0.473 (0.027)**	0.469	0.892 (0.031)	0.906	**0.113 (0.007)**	**0.116**
	KNN	0.606 (0.028)	0.663	0.350 (0.020)	0.378	0.742 (0.042)	0.818	**0.128 (0.008)**	**0.130**
	NB	0.576 (0.044)	0.558	0.417 (0.035)	0.383	0.638 (0.055)	0.631	**0.092 (0.008)**	**0.099**
	DT	0.642 (0.041)	**0.671**	0.508 (0.037)	**0.529**	0.688 (0.051)	**0.719**	**0.082 (0.007)**	0.082

The best performance for each classifier metric is shown in bold, and we also report the standard deviation.

The results in Table 5 demonstrate a significant improvement in the ${\text{F}}_{2}$-measure and the *MI* ratio of the CMS dataset after introducing SNA metrics. Specifically, among the 7 classifiers in the CMS dataset, 5 classifiers outperformed those in the TD dataset during the testing phase in terms of ${\text{F}}_{2}$ score. These classifiers are RF, XGB, LR, SVM, and DT. Especially the XGB classifier, which saw an increase in ${\text{F}}_{2}$ score from 0.725 to 0.749 during the validation stage, surpassing other classifiers. Furthermore, it is worth noting that both KNN and NB have experienced some decline. However, it is important to highlight that in similar studies, KNN and NB are considered to be underperforming classifiers [19]. The results during the validation stage were obtained on a dataset that the model had never seen before, indicating that the TDP model has improved generalization ability after introducing the SNA metrics. Additionally, for the training stage, apart from RF and DT classifiers showing a slight decrease in ${\text{F}}_{2}$ score, all other classifiers demonstrated some performance improvement in both training and validation. This suggests that after introducing the SNA metrics, the model may have gained additional useful information to better fit the data and thus improve its performance.

On the other hand, in terms of the *MI* ratio, 6 out of 7 classifiers (except TD) achieved better performance on the test set (refer to the description of the *MI* ratio in Section 3.6, the smaller the *MI* ratio, the better the model performance). This indicates that SNA metrics can effectively improve the detection ratio of the model, which means that developers can increase cost-effectiveness and reduce economic losses.

For completeness, the results for precision and recall are also provided. As can be seen from Table 5, the recall on the test set for most classifiers in the CMS dataset, except for XGB, decreased compared to the TD dataset. In terms of precision, better performance on the test set was achieved by RF, XGB and DT classifiers. However, as mentioned in Section 3.6, what needs to be done is to consider both the recall and precision of the model. A decrease in recall or precision alone cannot be the standard for judging the good or bad of the model. The excellent performance of the classifier in the ${\text{F}}_{2}$-measure indicates that after introducing SNA metrics, a better balance between recall and precision can be achieved by each classifier, which is what is being pursued.

Due to our primary focus on ${\text{F}}_{2}$-measure and the *MI* ratio, the introduction of the new metric (SNA metrics) has significantly enhanced the overall model performance in both training and testing phases, thereby improving its generalization capability and predictive accuracy.

Of course, this is the result obtained by using the same classifier parameters as Tsoukalas *et al*. [19]. In order to more comprehensively evaluate the impact of introducing SNA metrics on the performance of each classifier, *Grid-search* [40] was used to fine-tune the hyperparameters of the CMS dataset, and the experiment was conducted again. Table 6 reports the fine-tuning results of the classifier parameters after hyperparameter tuning was performed. Table 7 presents the performance of each classifier on the CMS dataset after hyperparameter tuning. It is evident that the performance of LR, SVM, KNN, and NB remained unchanged after hyperparameter tuning due to their parameters remaining consistent before and after tuning. Upon closer examination of classifiers with adjustments in other parameters, it is apparent that the ${\text{F}}_{2}$ performance of two classifiers (XGB and DT) improved, while the RF classifier exhibited no significant change. Specifically, the ${\text{F}}_{2}$ performance of the XGB classifier on the test set increased from 0.749 prior to hyperparameter tuning to 0.772, indicating an enhancement in prediction performance as a result of hyperparameter tuning. Conversely, for the RF classifier, there was minimal change as its ${\text{F}}_{2}$ performance decreased from 0.766 before tuning to 0.764 post-tuning. However, upon further examination of other performance metrics, the recall of RF showed improvement (from 0.828 to 0.867), albeit at the expense of a decrease in precision and model detection ratio. Since ${\text{F}}_{2}$ is utilized as the objective function for hyperparameter tuning, it is expected that the tuned model will give priority to recall. The decision of whether to trade off a certain degree of precision and model detection efficiency for higher recall is contingent upon the company’s discretion. Some companies may be willing to incur substantial costs in order to detect as many high-TD modules as possible, thereby mitigating potential economic losses in the future. Similar trade-offs were observed with DT and XGB classifiers, which also sacrificed some precision and model detection ratio post-tuning in exchange for improved metrics and recall. Overall, both before and after hyperparameter tuning, the combined metric suit (CMS) demonstrated enhanced model performance across seven categories compared to the singular use of TD - related metrics in most classifiers. KNN and NB classifiers were exceptions, which highlights the efficacy of SNA metrics within the TDP model. Furthermore, our research results confirmed the underperformance of KNN and NB classifiers—a sentiment echoed by previous studies (e.g., Ref. [19]).

**Table 6:** Classification models of the experimental setup.

Classification Model	Best Tuning Parameters
Logistic Regression (LR)	penalty=’none’, solver=’lbfgs’
Naive Bayes Classifier (NB)	N/A
Decision Tree (DT)	criterion=’gini’, max depth=5
k-Nearest Neighbor (KNN)	n neighbors=8
Support Vector Machine (SVM)	kernel=’rbf’, C=1
Random Forest (RF)	n estimators=100, criterion=’entropy’, max depth=10
XGBoost (XGB)	n estimators=10, booster =’gbtree’

**Table 7:** Evaluation results for all classifiers after hyper-parameter tuning.

		$F_{2}$		Precision		Recall		MI		
	Model	Validation	Test	Validation	Test	Validation	Test	Validation	Test	Cluster
CMS	RF	0.761 (0.023)	0.764	0.544 (0.026)	0.518	0.847 (0.034)	0.867	0.094 (0.007)	0.101	A
	XGB	0.762 (0.026)	0.772	0.533 (0.031)	0.528	0.855 (0.036)	0.872	0.097 (0.007)	0.099	A
	LR	0.767 (0.027)	0.767	0.482 (0.032)	0.475	0.901 (0.031)	0.906	0.113 (0.008)	0.114	A
	SVM	0.757 (0.024)	0.764	0.473 (0.027)	0.469	0.892 (0.031)	0.906	0.113 (0.007)	0.116	A
	KNN	0.606 (0.028)	0.663	0.350 (0.020)	0.378	0.742 (0.042)	0.818	0.128 (0.008)	0.130	C
	NB	0.576 (0.044)	0.558	0.417 (0.035)	0.383	0.638 (0.055)	0.631	0.092 (0.008)	0.099	D
	DT	0.739 (0.028)	0.734	0.431 (0.036)	0.422	0.903 (0.036)	0.901	0.127 (0.012)	0.128	B


The results for the other three classifiers indicate that, except for the RF classifier, the performance of the other two classifiers (XGB and DT) has improved in terms of ${\text{F}}_{2}$ score. In particular, the XGB classifier’s ${\text{F}}_{2}$ performance during testing has increased from 0.749 before parameter tuning to 0.772, indicating that parameter tuning has enhanced the predictive performance of the XGB model. As for the RF classifier, its ${\text{F}}_{2}$ performance decreased from 0.766 before tuning to 0.764, with almost no change; however, when considering other performance measures, RF’s recall rate has improved (from 0.828 to 0.867), at the expense of precision and *MI* ratio decreasing. Since ${\text{F}}_{2}$ was used as the objective function for parameter tuning, it can be foreseen that the tuned model will have a greater inclination towards improving the recall rate. Whether sacrificing a certain level of precision and model detection efficiency for higher recall rate depends on company decisions; some companies may prefer to invest significant costs in detecting as many high TD modules as possible to mitigate potential future economic losses. For both DT and XGB classifiers alike, they have sacrificed a certain level of precision and model *MI* ratio after tuning in exchange for improvements in measures and recall rates.

Among the five learning algorithms that demonstrated enhanced ${\text{F}}_{2}$ performance, Wilcoxon analysis revealed p-values below 0.05 for all models except the Random Forest approach.This discrepancy may arise from RF’s built-in feature selection via out-of-bag error estimation, which could reduce the marginal contribution of SNA metrics relative to existing baseline features. The null hypothesis was consistently rejected across experimental evaluations (p<0.05), confirming measurable performance disparities between different dataset partitions. This outcome corroborates our hypothesis about feature compatibility exerting measurable influence on predictive effectiveness, indicating that technical debt models achieve optimal performance when test data characteristics align with their training distributions.

**Fig 2:**
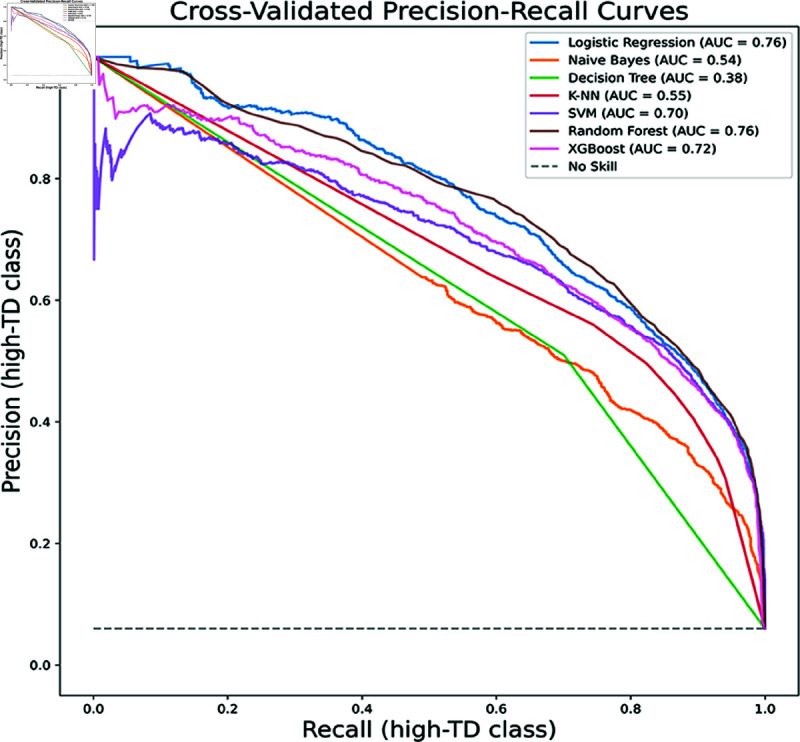
Cross-validated precision-recall curves (TD). The Precision-Recall curve for the TD dataset shows the tradeoff between precision (*y*-axis) and recall (*x*-axis) across probability thresholds, derived from 10-fold cross-validation.

**Fig 3:**
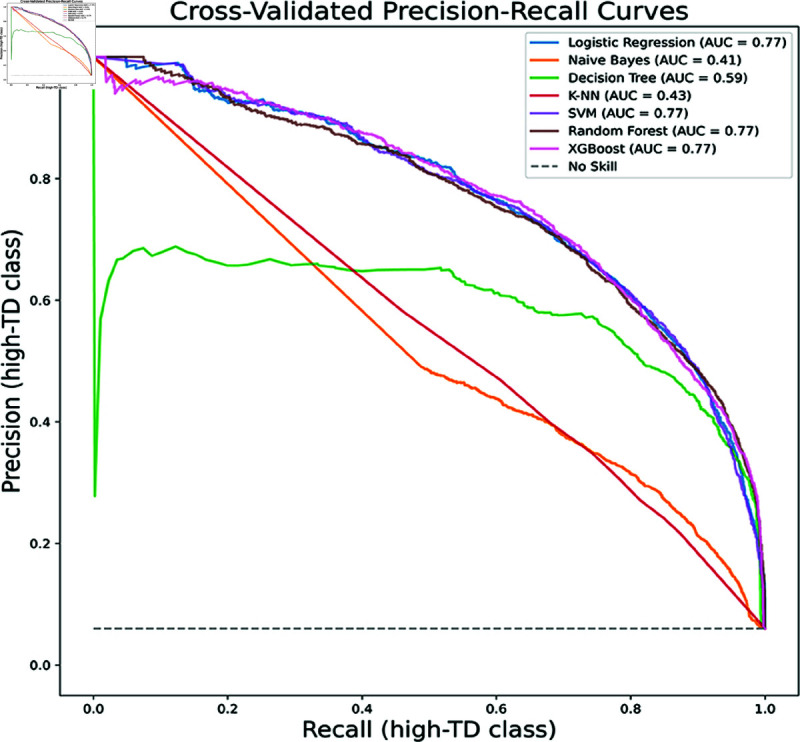
Cross-validated precision-recall curves (CMS). The Precision-Recall curve for the CMS dataset shows the tradeoff between precision (*y*-axis) and recall (*x*-axis) across probability thresholds, derived from 10-fold cross-validation.

However, comprehensive model evaluation requires interpretation through multiple metrics. We have consequently constructed precision-recall (PR) curves, which are more appropriate than other curve types for imbalanced datasets like ours (with a high-TD to non-high-TD module ratio of 1:15). Each classifier’s Area Under the Curve (AUC) value quantifies its threshold-agnostic performance, where higher AUC indicates better precision-recall balance, validating optimization effectiveness. Fig 2 and 3 display AUC values for models trained on the TD and CMS datasets respectively. The AUC results corroborate our experimental findings, showing improvements for all classifiers except KNN and NB after incorporating SNA metrics, particularly notable for DT’s AUC increase from 0.38 to 0.59.

These results demonstrate that CMS metrics (combining SNA and TD indicators) generally outperform standalone TD metrics across classifiers. This enhancement likely stems from SNA metrics supplementing critical system information or synergistically capturing broader software characteristics with TD metrics, analogous to how SNA complements code metrics in software defect prediction (SDP). To verify our findings and assess feature set informativeness, we implemented the PCA method from Long *et al*. [22], measuring how many principal components (PCs) each metric set requires to capture 95% data variance – fewer PCs indicate higher information density.

**Fig 4:**
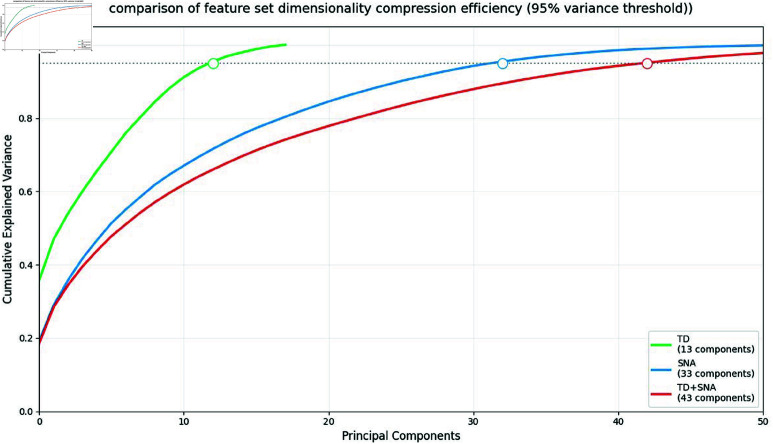
PCA cumulative variance plot. The PCA cumulative variance plot is a graphical representation that illustrates the accumulated explained variance ratio (*y*-axis) versus the number of principal components (*x*-axis).

Fig 4 illustrates cumulative variance curves, where the x-axis represents PC counts and y-axis shows explained variance. Key observations: TD metrics (green curve) need 13 PCs, indicating lower information density; SNA metrics (blue curve) require 33 PCs, demonstrating higher information content; CMS combined metrics (red curve) use 43 PCs – less than the additive total (46 PCs for 13 + 33), confirming metric complementarity. The CMS curve’s rightward shift reveals superior variance explanation per PC count.

This analysis concludes that TD-SNA integration achieves more efficient dimensionality reduction through complementary information synergy. Their combination creates richer feature representations, establishing merged TD/SNA metrics as a superior strategy for technical debt prediction models. Future research should prioritize such metric fusion approaches.

### 4.2 Which classifier performs best on TDPSN models?

As shown in Table 7, in terms of the validation phase, LR achieved the highest ${\text{F}}_{2}$ score of 0.767, followed by XGB and RF classifiers. On the other hand, the XGB classifier obtained the highest ${\text{F}}_{2}$ score of 0.772 in the testing phase. Following closely behind is RF with a score of 0.768 (the highest ${\text{F}}_{2}$ scores before and after tuning were selected). Then there are LR, SVM, and DT with scores ranging from 0.734 to 0.767. It is also noted that compared to the results in the validation phase, the performance of classifiers has even improved. To avoid the inflation problem caused by comparing multiple classifiers at the same time, we used the SK (Scott Knott) [44] algorithm to perform statistical hypothesis testing. Compared with other hypothesis testing methods, the SK algorithm generates clusters of classifiers with similar performance and sorts the clusters of classifiers according to performance. We input the ${\text{F}}_{2}$ scores of each classifier in the 3x10 cross-validation process, and finally the SK algorithm generated four clusters of classifiers with similar performance. The performance of the classifiers is ranked from best “A” to worst “D.” We report the results of the SK algorithm in the last column of Table 7. As we can see, RF, XGB, LR, and SVM are classified into the best classifier cluster, which means that they have similar prediction performance, which is also consistent with our previous experimental results. In addition, it further proves that KNN and NB can be considered the worst choices.

Although the ${\text{F}}_{2}$ measure is considered one of the primary performance metrics, it is important to consider other metrics in order to make the best choice. As shown in Table 7, DT, LR, and SVM exhibit the highest recall during the validation phase, with scores of 0.903, 0.901, and 0.892 respectively. Following closely are XGB and RF, while KNN and NB perform poorly in terms of recall and rank last. This trend generally holds true in the test phase as well; LR and SVM show improved recall compared to the validation phase, both reaching a score of 0.906. However, when it comes to precision, only RF and XGB classifiers have precision scores exceeding 0.5 during both validation and testing phases; all other classifiers fall below this threshold at between 0.3 to 0.5 range which may significantly increase the number of predicted false positives (${\text{FP}}_{S}$) since a precision score below 0.5 will result in more ${\text{FP}}_{S}$ than true positives (${\text{TP}}_{S}$) [19]. Considering the results from Table 6 before and after parameter tuning reveals that only three classifiers (RF, XGB, and DT) have precisions exceeding 0.5. While we prioritize recall over precision, this does not mean that we can disregard precision altogether; rather we aim for a model with moderate precision along with high recall rate.

To supplement the comparison of the above classifier performance metrics,Precision-Recall (precision-recall) curves are plotted, precision-recall curves are a better choice for unbalanced datasets (the ratio of high-TD modules to non-high-TD modules in the dataset we use is about 1:15). We also provide the area under the curve (AUC) value for each classifier, summarizing their performance at different thresholds. The higher the AUC value, the higher the recall and precision. As we can see from Fig 3, the curves of RF, XGB, LR, and SVM classifiers are closer to the top right corner, almost reaching the point of coincidence. This means that compared to other classifiers, they can achieve similar recall scores by sacrificing lower precision. In addition, the AUC values of these four classifiers are all 0.77, tying for first place. This is followed by DT (0.59), KNN (0.43), and NB (0.41). This is also consistent with our previous experimental results. The four best clustering classifiers obtained by the SK algorithm are also in the same echelon in terms of AUC performance. The horizontal dotted line below represents a noskill classifier, which is a classifier that predicts that all instances belong to the positive class. Its y-value is 0.067, which is equal to the ratio of high-TD modules to non-high-TD modules in the dataset.

Regarding the second major performance metric, the *MI* ratio, by examining the test phase scores in Table 7 and 8, we can find that the XGB classifier has the best overall performance. It achieved the lowest *MI* ratio (0.064) before hyperparameter tuning and tied for the highest score (0.099) with the NB classifier after hyperparameter tuning. However, considering that the NB classifier’s precision is less than 0.5 and its recall is the lowest among all classifiers (0.631), this means that it cannot effectively identify high-TD modules. Therefore, the XGB classifier’s performance in terms of *MI* ratio can be said to surpass all other classifiers. The RF classifier also performed well in this regard, ranking second and third among all classifiers in terms of *MI* ratio before and after hyperparameter tuning, respectively, demonstrating stable performance.

**Table 8:** Evolution results of classifiers for different metrics.

		$F_{2}$		Precision		Recall		MI	
TH Metrics	Model	Validation	Test	Validation	Test	Validation	Test	Validation	Test
EN	RF	0.540	0.558	0.329	0.340	0.644	0.665	0.118	0.117
	XGB	0.537	0.557	0.305	0.333	0.663	0.670	0.131	0.121
GN	RF	0.598	0.617	0.327	0.341	0.755	0.773	0.139	0.136
	XGB	0.580	0.596	0.292	0.302	0.771	0.788	0.159	0.157
SNA	RF	0.513	0.525	0.326	0.335	0.600	0.611	0.111	0.109
	XGB	0.498	0.527	0.311	0.313	0.587	0.635	0.113	0.122
Size	RF	0.729	0.732	0.445	0.422	0.892	0.892	0.121	0.127
	XGB	0.734	0.743	0.406	0.398	0.919	0.931	0.136	0.140
Evolution	RF	0.662	0.679	0.442	0.449	0.757	0.778	0.103	0.104
	XGB	0.671	0.689	0.384	0.382	0.827	0.862	0.130	0.135
OM	RF	0.699	0.693	0.437	0.420	0.823	0.828	0.113	0.118
	XGB	0.670	0.683	0.363	0.377	0.853	0.857	0.142	0.136
TD	RF	0.756	0.766	0.573	0.553	0.823	0.847	0.087	0.092
	XGB	0.753	0.759	0.459	0.456	0.899	0.911	0.118	0.120
CMS	RF	0.761	0.764	0.544	0.518	0.847	0.867	0.094	0.101
	XGB	0.762	0.772	0.533	0.528	0.855	0.872	0.097	0.099
SM	RF	0.758	0.760	0.497	0.520	0.860	0.872	0.100	0.105
	XGB	0.759	0.761	0.519	0.519	0.857	0.862	0.100	0.099

Based on the above results, we can easily conclude that XGB is the best classifier in our study, consistently outperforming other classifiers at all stages. The RF classifier can be considered as the second choice, also demonstrating excellent predictive performance and widely regarded as one of the best classifiers in other studies (e.g., Ref. [19]). Such results are not surprising at all, as more complex algorithms (such as RF, XGB, and SVM) perform better than simpler ones (such as DT or NB). This may also be due to the presence of nonlinear underlying relationships in our dataset, leading to better performance of these classifiers.

### 4.3 Are SNA metrics more effective than TD metrics?

Table 8 presents classifier evaluation results across different metric sets, with detailed combinations described in Section 3.3. Given our focus on optimizing for ${\text{F}}_{2}$-score during parameter tuning, the CMS dataset achieves the highest ${\text{F}}_{2}$-values in both training and testing phases, outperforming all other metric sets. The TD and SM datasets follow closely. Notably, the SM dataset – derived from CMS via feature selection – secures the second-best ${\text{F}}_{2}$-score with XGBoost, marginally trailing CMS. Contrastingly, RF classifier shows comparable performance between SM and TD sets: SM slightly outperforms TD during validation (0.758 vs. 0.756) but underperforms in testing (0.761 vs. 0.766). This counterintuitive outcome challenges theoretical expectations, as SM’s removal of highly correlated features should enhance predictive performance. Instead, it lags behind CMS and even TD in specific cases, aligning with findings by [22] that standalone SNA metrics show limited defect prediction capability but improve performance when combined with code metrics.

Further analysis from Table 8 reveals poor ${\text{F}}_{2}$-scores for the full SNA set, underperforming even EN and GN metrics. This suggests potential masking effects from internal feature correlations or Simpson’s paradox [45], where beneficial individual metrics combine detrimentally. Remarkably, the CMS set (SNA+TD integration) achieves optimal results, demonstrating synergistic complementarity.

In order to better observe the performance differences of models trained based on different feature sets, the indicators in this paper are skewed and have unequal variances. Therefore, we used the non-parametric Mann-Whitney U test [46]. And we tested our hypothesis at a confidence level of 95% (a= 0.05). This method mainly obtains a U statistic and the corresponding p-value. The p-value is used to determine whether there is a significant difference between the two groups of data. If the p-value is less than the pre-set significance level (0.05), the null hypothesis is rejected, indicating that there is a significant difference in the distributions of the two groups of data; otherwise, the null hypothesis cannot be rejected.

Three sets of comparative experiments were conducted, with the indicator groups being “TD-related metrics + GN metrics,” “TD-related metrics + EN metrics,” and “TD-related metrics + SNA metrics” respectively, and compared them with the model using only TD-related metrics. We used the Mann-Whitney U test to compare the performance differences of the models. Table 9 shows the results of the Mann-Whitney U test. We found that except for the “TD + EN” indicator group, the p-values of the other two groups of experiments were all less than 0.05. In particular, for the combination of “TD + SNA,” its p-value was 0.0328, which indicates that there is a significant difference between the results of the two models. This further validates the experimental results of the previous chapter of this paper, that is, the SNA metrics can improve the existing TD-related indicator system, thereby enhancing the predictive performance of the model.

**Table 9:** Mann-Whitney U test results of TD metrics and SNA metrics.

Comparison metrics	Statistic	P-value	P-value< 0.05
TD + GN & TD	558	0.0460	Yes
TD + EN & TD	450	0.5029	No
TD + SNA & TD	575	0.0328	Yes

Based on the above results, we cannot clearly state that the SNA metrics are superior to the TD-related metrics, nor can we consider that the SNA metrics are better than the GN and EN metrics. However, we can draw a conclusion that when the SNA metrics are combined with the TD-related metrics, they can complement the information of the TD-related metrics, thereby improving the predictive ability of the model.To verify our conjecture, we used PCA analysis again to quantify the ability of different indicator sets to capture TD information. Table 10 shows the median number of components required for each indicator set to account for 95% of the data variance information. The larger the number of components, the greater the amount of information contained in the indicator set. We can find that CMS and SM have captured the most information. This also supports our hypothesis, indicating that the combined indicator sets (CMS and SM) have added additional information to the TD metrics. On the other hand, feature screening may have weakened some of the information, resulting in a decrease in the performance of the model (the amount of information in the SM dataset is less than that in the CMS dataset).

**Table 10:** The median number of components required by each metric family to account for 95% of data variance information.

Metric family	TD	SNA(EN/GN)	CMS	SM	TD+GN	TD+EN
Median number	13	33 (21/17)	43	35	32	28

### 4.4 Can diverse metric combinations influence the performance of TDPSN models?

Table 11 presents the results of our experiments on the combinations of SNA-related metrics and TD-related metrics. We followed the baseline method in Section 3.5 for our experiments, using the best classifiers XGB and RF obtained in Section 4.2 as our research classifiers. The detailed classification of SNA metrics and TD metrics refers to the metric system in Section 3.3. Considering that the number of Duplication, Complexity, Coupling, and Cohesion metrics are much smaller than Evolution and Size metrics (3 at most, 1 at least), and we found in subsequent experiments that these four types of metrics have poor performance when used alone, so we combine these four types of metrics to form a TD metrics combination with 6 TD metrics, which we call the OM metrics.

**Table 11:** Evaluation results of classifier after metrics combination.

			$F_{2}$		Precision		Recall		MI	
TH Metrics	SNA Metrics	Model	Validation	Test	Validation	Test	Validation	Test	Validation	Test
Size	SNA	RF	0.745	0.767	0.552	0.547	0.818	0.852	0.089	0.093
		XGB	0.763	0.762	0.517	0.500	0.867	0.877	0.101	0.105
	EN	RF	0.738	0.737	0.538	0.504	0.815	0.833	0.091	0.099
		XGB	0.746	0.757	0.488	0.482	0.861	0.882	0.106	0.110
	GN	RF	0.751	0.765	0.557	0.561	0.824	0.842	0.089	0.090
		XGB	0.761	0.756	0.501	0.486	0.876	0.877	0.106	0.108
Evolution	SNA	RF	0.583	0.597	0.517	0.504	0.604	0.626	0.070	0.075
		XGB	0.682	0.686	0.471	0.466	0.770	0.778	0.098	0.100
	EN	RF	0.570	0.580	0.508	0.522	0.589	0.596	0.070	0.069
		XGB	0.681	0.700	0.463	0.470	0.773	0.798	0.101	0.102
	GN	RF	0.617	0.685	0.495	0.521	0.658	0.744	0.080	0.086
		XGB	0.672	0.676	0.443	0.420	0.773	0.798	0.105	0.114
OM	SNA	RF	0.686	0.700	0.551	0.555	0.732	0.749	0.080	0.081
		XGB	0.708	0.738	0.471	0.469	0.812	0.862	0.104	0.110
	EN	RF	0.678	0.665	0.536	0.512	0.728	0.719	0.082	0.084
		XGB	0.696	0.703	0.460	0.433	0.800	0.833	0.105	0.115
	GN	RF	0.701	0.705	0.539	0.540	0.759	0.764	0.085	0.085
		XGB	0.717	0749	0.469	0.473	0.828	0.877	0.106	0.111
TD	SNA	RF	0.761	0.764	0.544	0.518	0.847	0.867	0.094	0.101
		XGB	0.762	0.772	0.533	0.528	0.855	0.872	0.097	0.099
	EN	RF	0.746	0.745	0.570	0.559	0.809	0.13	0.085	0.087
		XGB	0.753	0.768	0.520	0.521	0.849	0.872	0.098	0.101
	GN	RF	0.759	0.769	0.585	0.578	0.821	0.837	0.084	0.087
		XGB	0.764	0.772	0.522	0.552	0.856	0.877	0.100	0.101

From the results, it is evident that the Size metrics comprehensively outperforms the other two sets of metrics (Evolution metrics and OM metrics) when combined with the SNA metrics. Specifically, both RF and XGB achieve higher scores in ${\text{F}}_{2}$ when compared to their performance in other metrics combinations. Furthermore, the performance of the Size metrics in combination with the SNA metrics is comparable to that of the complete TD and SNA metrics combination. In fact, the ${\text{F}}_{2}$ score of the RF classifier even surpasses that of the complete TD and SNA metrics combination. Additionally, when combined with EN and GN metrics, the Size index also outperforms both sets of metrics in terms of ${\text{F}}_{2}$ scores, as well as demonstrating good performance in other performance metrics such as precision, recall, and *MI* ratio.

Based on the results, it is evident that Size-related metrics outperform other TD-related metrics. Specifically, when Size, metrics and Evolution metrics in TD metrics are combined with SNA-related metrics (SNA, EN and GN metrics), the performance of Size-related metrics is superior to other combinations of metrics across two classifiers in all nine experimental groups. Even when combined with SNA metrics, the RF classifier’s ${\text{F}}_{2}$ score exceeds that of “TD-related metrics + SNA metrics” combinations. Furthermore, upon observing other performance metrics (precision, recall, and *MI* ratio), Size-related metrics continue to demonstrate outstanding performance. This suggests that compared to other metrics, Size metrics has a significant impact on the performance of the TDP model as it can better capture key patterns or information related to TDP.

Results emphasize the superiority of Size-related metrics in TD identification. Combinations involving Size metrics and SNA metrics (including EN and GN) consistently dominate other TD metric combinations across classifiers and experimental groups, suggesting Size metrics effectively capture TD-related patterns. Mann-Whitney U tests (Table 12) reveal statistically significant differences (all p-values = 0) between Size-based combinations versus Evolution and OM metrics in 6 comparison groups.

**Table 12:** Mann-Whitney U test results of GN metrics and EN metrics.

Comparison metrics	Statistic	P-value	P-value< 0.05
GN + Size & EN + Size	590	0.0196	Yes
GN + Evolution & EN + Evolution	387	0.8261	No
GN + OM & EN + OM	642.5	0.0023	Yes
GN + TD & EN + TD	555	0.0412	Yes

Examining the performance of EN and GN metrics when combined with TD-related metrics: The RF and XGB classifiers each underwent four combination experiments. In three out of four combination experiments for RF classifier and two out of four for XGB classifier respectively, GN’s ${\text{F}}_{2}$ score exceeded that of EN. Particularly in experiments combining “OM-related metrics + EN/GN” and “TD-related metrics + EN/GN metrics” combinations where GN’s ${\text{F}}_{2}$ score surpassed EN’s on both XGB and RF classifiers. While we cannot definitively state that GN metrics outperforms EN metrics overall; however under certain circumstances within our project context at least there is evidence suggesting that GN metrics may exhibit better predictive performance than EN metrics within the TDP model framework.

Table 13 details comparison tests between GN and EN metrics in TD-related combinations. Mann-Whitney U tests show statistically significant superiority of GN in 3 out of 4 experimental groups (p<0.05), except when combined with Evolution metrics. This confirms GN’s enhanced predictive capability in most combination scenarios.

**Table 13:** Mann-Whitney U test results of Size metrics.

Comparison metrics	Statistic	P-value	P-value< 0.05
Size + SNA & Evolution + SNA	868	0.0000	Yes
Size + GN & Evolution + GN	895	0.0000	Yes
Size + EN & Evolution + EN	843	0.0000	Yes
Size + SNA & OM + SNA	822.5	0.0000	Yes
Size + GN & OM + GN	797	0.0000	Yes
Size + EN & OM + EN	825	0.0000	Yes

In conclusion, while CMS-trained models achieve the highest prediction performance, strategic feature selection and combination (particularly with Size-related metrics) can yield comparable or superior results. Future studies should prioritize GN over EN metrics and emphasize Size-related metrics for TDP, as global network metrics (GN) better characterize technical debt patterns than local network metrics (EN). For software maintenance practices, practitioners should prioritize high-size modules (e.g., complex modules) for TD remediation, as size-related factors significantly influence technical debt occurrence and system risk mitigation.

## 5 Discussion

From the above result, it can be observed that the SNA metrics indeed improves the existing TDP models. Prior to this study, there had been no similar research, so our study can be said to fill this gap. In RQ1, the TDPSN models constructed by CMS containing SNA metrics obtained better generalization performance. Furthermore, results from Q2 indicated that XGB stood out as the best classifier in our study, contrary to similar studies (e.g., Ref. [19]) that favored RF as the top classifier. XGB is acknowledged as the best classifier for this research, with RF performing closely behind XGB. Gong *et al*. [22] suggested that GN and EN metrics should be considered separately as they capture different software characteristics, and combining GN and EN metrics may weaken their individual signals. Their conclusion supports this viewpoint.

Therefore, in RQ3, we investigated the ability of different categories of metrics to identify Technical Debt (TD). The experimental results show that the predictive ability of Social Network Analysis (SNA) metrics is not significant when used alone. However, when SNA metrics are combined with TD-related metrics, they can complement the information of TD-related metrics, thereby improving the predictive ability of the model. Additionally, when we use a part of the SNA metrics, the predictive ability of the model is inferior to that when using all 64 SNA metrics. In RQ4, we also conducted similar experiments and combined different relevant metrics with TD-related metrics respectively, such as size-related metrics and evolution-related metrics, to avoid Simpson’s Paradox (This refers to a situation where combinations of different data may not lead to classification for each range. Perspective) [45]. Our results also indicate that, under certain combined circumstances, Graph Network (GN) metrics do outperform Entity Network (EN) metrics, and even outperform the complete set of SNA metrics. In some cases, the combination of "size-related metrics + SNA metrics" is superior to other indicator combinations. Therefore, we suggest that future work should not only consider the comprehensive impact of the complete set of metrics, but also take into account the individual impact of each category of metrics separately. For software maintenance practices, maintenance personnel should give priority to reviewing high-scale modules (such as complex modules), because the scale factor has the most significant impact on technical debt. Prioritizing the repayment of technical debt for such modules can more effectively reduce system risks.

**Threats to validity**: In constructing the SNA dataset, some modules were skipped during the extraction of SNA metrics, leading to a mismatch in the number of modules in our (combined metric suit) CMS dataset compared to Tsoukalas *et al*. [19]. However, as these missing modules constituted a small proportion, we opted to remove them. While Tsoukalas *et al*. [19] also removed modules that their software tool could not extract, this may influence our experimental outcomes. Furthermore, we utilized oversampling techniques in data preprocessing to improve the model’s performance on minority classes, potentially leading to a discrepancy between our balanced dataset and the actual distribution. Future work will concentrate on evaluating the applicability of our findings to data reflecting real distributions.In the SDP domain, Gong *et al*. [22] favoring SNA metrics over other software metrics typically utilize 60 or more SNA metrics, a pattern consistent with our experiment where we incorporated all 64 SNA metrics. However, this decision may impact the model’s generalization capability, potentially limiting the applicability of our findings to diverse projects. Moreover, our study solely contrasts SNA metrics with software metrics used in a public dataset, which could affect the generalization of our TDP models. Future research directions will involve validating the effectiveness of SNA metrics across a variety of projects and metric systems used in other TDP models.

## 6 Conclusion

While the SNA metric has been widely utilized and has sparked significant debate in other areas of software engineering, such as Software Defect Prediction (SDP) research, there is a lack of similar research in the field of TDP. Some studies (e.g., Ref. [22]) have found that in certain cases, combining SNA metrics with code metrics can be superior to using code metrics alone. Therefore, in this study, we extracted SNA metrics from 25 open-source Java projects and combined them with existing TD-related metrics to construct the TDP model as CMS, to investigate whether SNA metrics can improve existing TDP models. The experimental results show that after introducing SNA metrics, five out of seven classifiers perform better on the TDP model than when using TD-related metrics alone. Among them, XGB is the best classifier in our study, performing better on the TDPSN model than other classifiers. Therefore, we suggest that future research should consider using both SNA and TD-related metrics when constructing TDP models. In addition, we recommend not only considering composite SNA metrics but also separately considering EN and GN metrics within the SNA metrics.
